# Research on the Weld Position Detection for the T-Joints in Web-Core Sandwich Panels Based on Eddy Current Technology

**DOI:** 10.3390/s20092691

**Published:** 2020-05-08

**Authors:** Angang Wei, Baohua Chang, Fanyue Meng, Dong Du, Zandong Han

**Affiliations:** 1 Department of Mechanical Engineering, Tsinghua University, Beijing 100084, China; waa16@mails.tsinghua.edu.cn (A.W.); bhchang@tsinghua.edu.cn (B.C.); mfy17@mails.tsinghua.edu.cn (F.M.); 2 Key Laboratory for Advanced Materials Processing Technology, Ministry of Education, Beijing 100084, China

**Keywords:** web-core sandwich panels, T-joint welded from face-panel side, weld position detection, eddy current technology

## Abstract

Web-core sandwich panels have gained the popularity in various fields, especially aviation and shipbuilding, etc. Penetration welding was considered as an effective process to manufacture such a structure through a T-joint. To ensure the formation quality and mechanical properties of weld, the welding torch needs to be aligned with the T-joint position. However, it is difficult to locate the T-joint position (i.e., the position of core panel) because of the shielding of the face panels. This paper investigated the detection of T-joint position from the face panel side in web-core sandwich panels based on eddy current technology. First, we designed an experimental system for the weld position detection of T-joints from the face panel side. The relationships are investigated between the characteristics of the eddy current detection signal and the primary parameters of the detection system (including excitation frequency, coil outer diameter, and lift off distance) and the T-joint (including thickness of the core panel, gap distance, and thickness of the cover panel). Corresponding experiments were carried out with variable primary parameters, and the influence mechanism of the primary parameters on the detection results in terms of sensitivity and dynamic performance was elaborated to set up the theoretical basis for the detection. Finally, weld position detection experiments were carried out on TC4 titanium alloy T-joint specimens with 3 mm-thick face panel and 5 mm-thick core panel. Results showed that the maximum detection error was 0.482 mm, and the average error was 0.234 mm. This paper provided a possible technical solution to the automatic tracking problem for the welding of T-joints in the web-core sandwich panels.

## 1. Introduction

**Figure 1:**
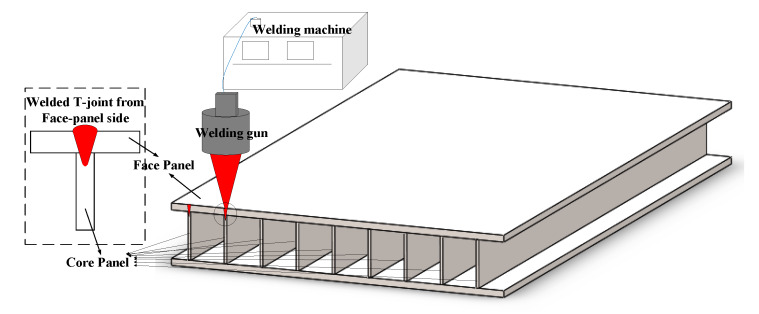
Welded T-joint from the face panel side in the web-core sandwich structure.

Web-core sandwich panels are kinds of lightweight structures with many advantages, such as the high strength and high stiffness to the weight ratio. The characteristics of the structure make it potential in many fields, such as aviation, aerospace, the marine shipbuilding industry, etc. [1,2,3,4]. The structure consists of two face panels separated by core panels that are aligned orthogonally to the faces (Figure 1). Welding is considered as an effective process to join the cover and core panel with T-joints. However, it is sometimes impractical to weld T-joints using conventional fillet welds due to the shielding of the face panels. Instead, the welding must be achieved from the face panel side to connect the face panel to the core panels (Figure 1) [5,6]. Many researchers have concerned the welded T-joints from the face panel side. The influence of weld width on the mechanical properties of laser-welded joints of hull steel CCS-B sandwich plates were analyzed based on the experimental investigation and finite element method [7]. Romanoff et al. [8] proposed a novel evaluation approach of the web-core sandwich structural response under patch load. Authors in [4,9] proposed a new method for the thermal elasto-plastic analysis for T-joint laser welding to reduce the computation time by employing shell element model replacing the solid element model. Yong [10] studied the effect of the laser process (such as laser power, spot diameter, etc.) on the weld formation quality in the in the welding of a sandwich structure made of dissimilar-metals of AZ31B magnesium alloy and 304 stainless steel at Taiyuan University of Technology. The mathematical model of the formation process of the porosity in the weld pool of the T-joint on steel sandwich structures was established in [11]. As far as the T-joints penetration welding in sandwich structures is concerned, it is important to detect the weld position to ensure the quality of joint. For example, when the deviation of welding torch and the weld position becomes out of range, the connection strength of the welded joint could not be guaranteed. Weld position detection could remain the good quality of weld. Accordingly, it is necessary to develop an effective method for detecting the weld position in web-core sandwich panels.

At present, the visual method is widely utilized for weld detection in various weld forms [12,13,14,15,16]. However, it is impossible to observe the weld using visual methods due to the characteristics of sandwich structures. This special welding mode for T-joints challenges novel weld position detection methods. Accordingly, several researchers have made efforts around the detection technologies to detect this special joint. A control system for automatic seam tracking for T-joint FSW based on weaving was presented [17,18], in the proposed technology, the tool weaves back and forth perpendicular to the joint line during welding in order to discover the position of maximal axial force, which is shown to be the center of the weld. The information of deviation status is acquired by the metallic vapor plume, which is influenced by instability or rupture of keyhole while the torch deviates from the weld seam [19]. Detection method based on backscattered X-rays was proposed and carried out on a nickel-based alloy specimen [20,21], the method utilized the X-ray scanning the weld to determine the center of weld where the backscattered X-rays intensity reached peak. The literature [22] further explored the weld position detection method based on backscattered X-ray, an analytical model was established to calculate and optimize the parameters of detection system and several experiments were carried out on aluminum alloy specimen in terms of detection accuracy and dynamic performance.

However, existing weld detection technologies of T-joint in web-core sandwich structures could be improved. The method based on axial force is only available in specific welding process. The method based on vapor image may fail to establish the precise relationship between the characteristics of the detection signal and the deviation of the weld seam in some conditions where the molten pool remains inside the specimen due to a large-thickness core panel. To achieve sufficient contrast and sensitivity of signals acquired at the weld position, the X-ray-based methods restrict the dynamic performance of detection, and the safety issues need be dealt with in the X-ray environment.

Accordingly, we developed a weld position detection method for the T-joint based on eddy current technology and utilized the proposed method to perform static detection of weld positions along the cross section of the T-joint in aluminum alloy specimen [23]. In this paper, we will further investigate the relationship between the characteristics of the eddy current detection signal and the primary parameters of the detection system (including excitation frequency, coil outer diameter, and lift off distance) and the primary dimensional parameters of weld (including thickness of the core panel, gap distance, and thickness of the cover panel). Detection experiments with variable primary parameters are carried out. Based on the analysis of the experimental data, the influence mechanism of the primary parameters on the detection results in terms of sensitivity and dynamic performance of detection is analyzed. Finally, tracking experiment is carried out on a TC4 titanium alloy specimen with 3 mm-thick face panel and 5 mm-thick core panel.

## 2. Detection Method

**Figure 2:**
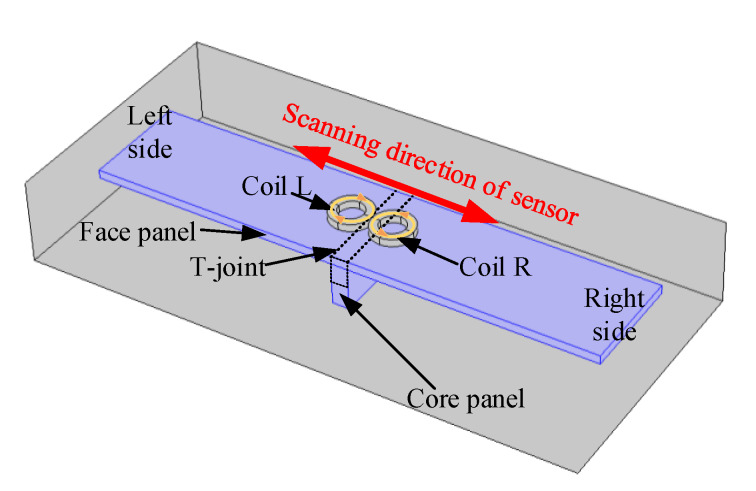
The scheme for eddy current sensor scanning to locate the center of core panel.

As shown in Figure 2, the proposed eddy current sensor consists of two separated coils with the same parameters and the same alternating excitation signal, the two coils are winded in the opposite direction, one coil is winded clockwise while the other one is winded anticlockwise from the top view. Two separated coils named as coil L and coil R are arranged side by side symmetrically about the T-joint to scan the specimen along the cross section of a T-joint weld. When the T-joint enters the sensitive region of eddy current sensor, the change of the induced current in specimen would result in alteration of magnetic flux passing through the two separated coils, which lead to the change of the equivalent impedance and the output voltage signals *V_l_* and *V_r_* of two separated coils. The difference Δ*V* of *V_l_* and *V_r_* is taken as the output signal of the eddy current sensor. The feature point, which represents the center position of the T-joint (i.e., the center position of the core panel), could be determined from the amplitude curve of sensor output signal.

**Figure 3:**
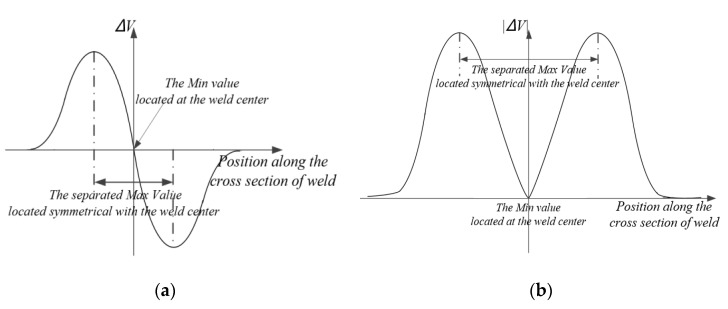
The sensor output signal during single scanning period (**a**) and the amplitude curve of sensor output signal (**b**).

Figure 3a shows the voltage difference of output voltage of coil L and coil R during a scanning of the T-joint weld from left to right side. Firstly, T-joint approached the magnetic field excited by coil R, the induced eddy current of core panel merely reduced the amount of magnetic flux passing through coil R, causing the decrease of equivalent impedance and increase of output voltage *V_r_*. The voltage difference was less than 0 and the amplitude of voltage difference gradually increased. Then, the output voltage of coil L began to increase and the amplitude of the voltage difference decreased as the T-joint entered the magnetic field excited by the coil L. When the coil L and coil R were completely symmetrical about the seam center (the center of core panel), the induced eddy current of specimen resulted in a same effect on the coil L and coil R. The impedances were equivalent in the two coils and the output voltages *V_l_* and *V_r_* of coil L and coil R remained synchronized. The amplitude of voltage difference would be reduced to zero. The *V_l_* increased and the *V_r_* decreased when the T-joint moved out of the magnetic field excited by coil R, and the output amplitude would gradually increase to a maximum value as the T-joint entirely moved out of the field. Finally, the amplitude of voltage difference decreased to zero while the T-joint kept moving out of the magnetic field excited by coil L.

Figure 3b shows the amplitude of sensor output signal when completing a single scanning of the weld. Considering that the parameters of coil L and coil R were consistent and the arrangement of two separated coils and the T-joint were symmetrical about the seam center, the signal amplitude was symmetrical about the center of weld.

As shown in Figure 3b, there are two methods to determine the feature points, which represent the center of weld: (1) Minimum value method. The minimum value acquired between two separated maximum values indicates the center position of weld. (2) Max-to-max center method. The two maximum amplitude values acquired in single scanning period are located symmetrical about the center position of weld, the seam center could be determined by the center position between the two maximum amplitude values.

## 3. Characteristic Analysis of the Proposed Eddy Current Sensor 

**Figure 4:**
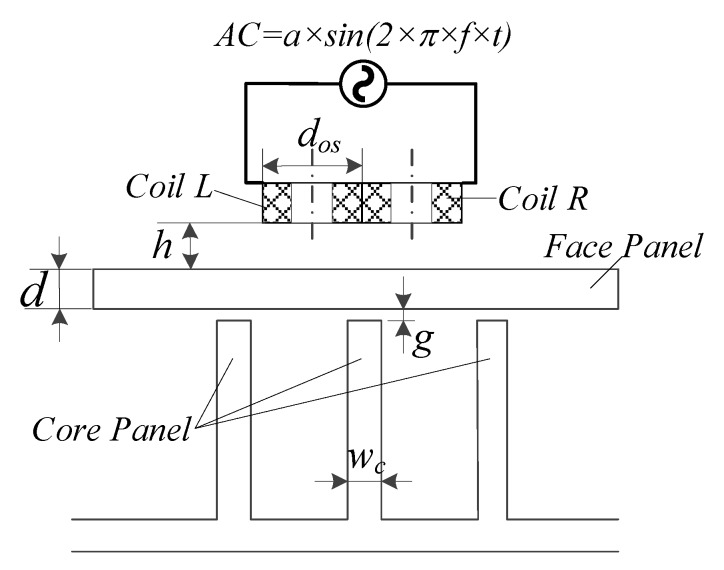
Primary influence factors of the eddy current sensor.

As shown in Figure 4, the primary factors affecting the output signal of the proposed eddy current sensor and the capability of detection system are considered. These include the excitation frequency *f*, the coil parameters (especially the outer diameter of coil *d_os_*), lift-off distance *h* (distance between the bottom surface of the coil and the specimen), and the dimension of weld (the thickness of core panel *w_c_*, the gap distance *g*, thickness of cover panel *d*). As shown in Equation (1):

*Z* = *F* (*f*, *d_os_*, *d*, *w_c_*, *g*, *h*), (1)

where *Z* is the coil impedance of eddy current sensor, *f* is excitation frequency, *d_os_* is coil outer diameter, *d* is thickness of cover panel, *w_c_* is thickness of core panel; *g* is the gap distance; and *h* is the lift off distance.

In this paper, a series of experiments about the influence of the above factors on the output signal of proposed sensor were carried out on the T-joint of a titanium alloy specimen. The experimental results were analyzed together with simulation results.

### 3.1. Excitation Frequency f

The key to detect the T-joint position based on eddy current technology lies in locating the core panel under the face panel. The excitation field by the eddy current sensor must be capable of penetrating through the face panel and exciting eddy current in the core panel whose current intensity is sufficient to be detected by the sensor. Considering the skin effect, the excitation frequency had a predominated effect on the depth of penetration of eddy current sensor and detection sensitivity.

In order to determine the optimal excitation frequency for the sensor to obtain the highest sensitivity, a series of experiments were carried out with different exciting frequencies from 20 to 40 kHz for the lift off distance of 0 mm. The specimen consisted of a 3 mm-thick TC4 cover panel and a 5 mm-thick TC4 core panel, and the gap distance was 0 mm. The outer diameter of the sensor coils was 20 mm.

**Figure 5:**
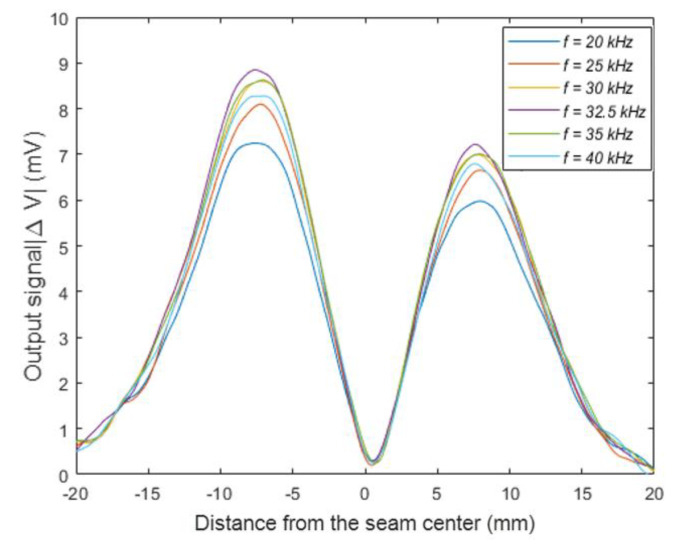
The sensor output signal with a different excitation frequency.

As shown in Figure 5, when the exciting frequency increased from 20 to 32.5 kHz, the output signal became more sensitive to the distance from the seam center. When the frequency was increased to 32.5 kHz, the change in output became the most significant. On the contrary, for the frequency higher than 32.5 kHz, the output signal decreased. Therefore, a frequency of 32.5 kHz was decided to be the optimal exciting frequency of a given specimen.

**Figure 6:**
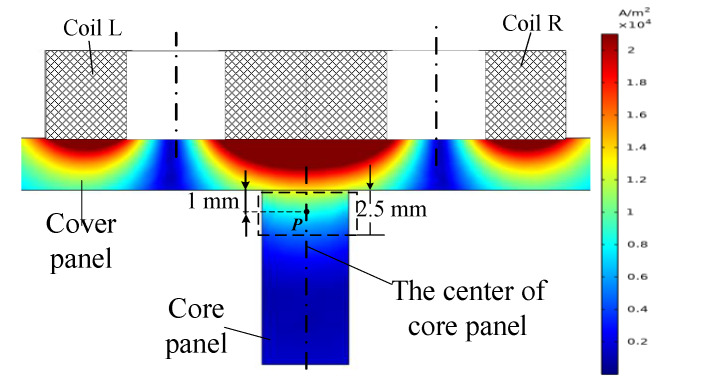
Distribution of the eddy current density in the specimen with excitation frequency *f* = 32.5 kHz.

**Figure 7:**
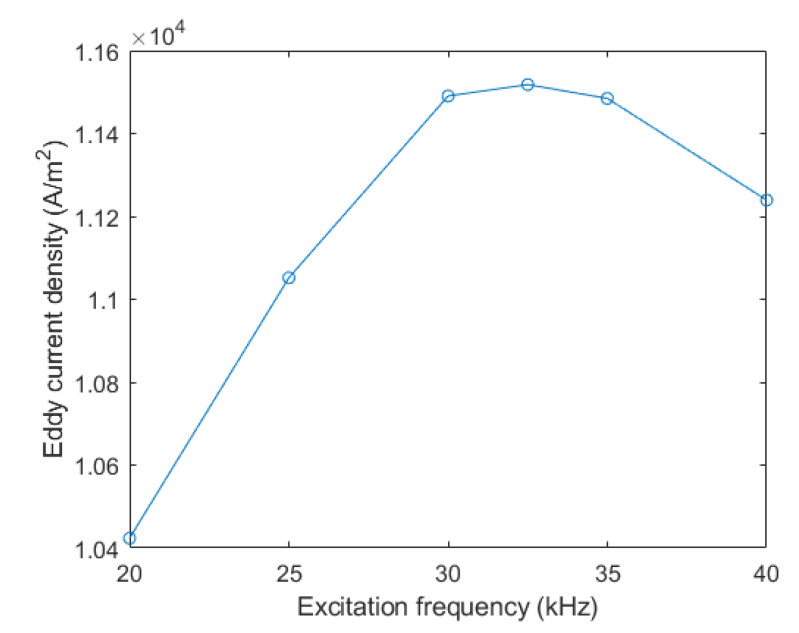
The eddy current density of point *P* under a different excitation frequency.

This paper simulated the distribution of eddy current density in the specimen with different excitation frequencies varying from 20 to 40 kHz based on COMSOL multiphysics. The parameters of the excitation signal and materials are listed as Table 1 and Table 2. The other simulation parameters were consistent with above experimental parameters. Figure 6 shows the distribution of eddy current density along the cross section of specimen while the center of sensor was aligned to the center of core panel. A point *P* was located at the center of core panel at a distance of 1 mm from the upper face of core, and the eddy current density at this point was capable of representing the eddy current distribution in the upper area of the core panel. Figure 7 shows the eddy current density at point *P* under variable excitation frequencies. Since the proposed sensor is a self-comparative sensor, the output signal of the sensor was determined by the eddy current density in the upper area of the core panel. The higher excitation would not produce a larger output signal of sensor. While the excitation frequency increases, the eddy current density near the sensor would increase and the eddy current density also decays faster along the depth. When it reaches the core panel, the eddy current density will be smaller than that of the lower excitation frequency. As shown in Figure 7, with the increase of the excitation frequency, the eddy current density would firstly increase and then decrease. The density was the largest at the excitation frequency of 32.5 kHz. The impedances of the coil L and coil R were separately 112.5 Ω and 113.6 Ω. The difference between impedances of two coils also reached the peak simultaneously.

There is an inverse relationship between the penetration depth of the eddy current sensor and the square root of excitation frequency [24]. As shown in Equation (2), for a specimen made of given material, the higher excitation frequency leads to the smaller penetration depth of sensor. On the other hand, the induced current density is proportional to the frequency within the range of penetration depth of sensor (see the Figure 6). The sensitivity of the proposed detection method of the T-joint is determined by the cumulative intensity of the eddy current density in the core panel in the depth and radial directions. Therefore, the appropriate excitation frequency should be determined by the tradeoff between the sufficient penetration depth in the cover panel and the enough induced current density of in the core panel along the depth (the upper area of the core panel). When the cumulative intensity of eddy current density in the upper area of the core panel (sensitive volume of sensor) reaches the maximum and the sensitivity of proposed detection method at this time would be best.

(2) $$h_{p}=\sqrt{\frac{1}{\pi \mu_{0}\mu_{r}f\sigma }}$$

where *h_p_* is the standard penetration depth of sensor, *f* is excitation frequency, *σ* is electrical conductivity of specimen, and *u*_0_ is the permeability of vacuum, taken as 4π × 10^−7^ h/m. *u_r_* is the relative permeability.

The electrical conductivity σ of titanium was 7.047 × 10^5^ s/m and the relative permeability *u_r_* was 1. These parameters were substituted into Equation (2) and the standard penetration depth *h_p_* was 3.24 mm. This depth is approximately equal to the thickness *d* of the cover panel.

(3) $$h_{p}\approx d$$

Several experiments were performed on the aluminum alloy specimen at different excitation frequencies [23]. The specimen consists of a 3 mm-thick cover panel and 10 mm-thick core panel of the 6061 aluminum alloy. The experiments are carried out from 500 to 2000 Hz with the interval of 500 Hz. The experimental result indicates that the detection sensitivity was highest when the excitation frequency was 1500 Hz. The electrical conductivity σ of 6061 aluminum alloy was 2.265 × 10^7^ s/m, these parameters were substituted into Equation (2), the standard penetration depth *h_p_* was 2.52 mm, which was also close to the thickness of the cover panel.

The sensitivity of the proposed detection method of the T-joint was determined by the cumulative intensity of eddy current density of sensitive volume in the core panel. With the increase of the excitation frequency, the eddy current density in the core panel would first increase and then decrease. Thus, the optimal excitation frequency could be acquired to penetrate the cover panel and induce sufficient eddy current density of sensitive volume in the core panel. According to Equations (2) and (3), an approximate optimal frequency could be estimated, and then the optimal frequency can be obtained by testing under multiple frequencies around the estimated approximate frequency.

### 3.2. Coil Outer Diameter d_os_

As described in Section 2, the seam center could be determined by the center of two locations with the maximum amplitude values. With the spacing of two separated maximum value decreased, the shorter scanning distance along the cross section of T-joint in single scanning period would be required to determine the center position of weld. The dynamic performance of the proposed detection method would be improved. This paper studies the influence of variable coil outer diameters on the detection results.

In order to analyze the influence of variable coin outer diameters on the sensor output signal, several experiments were carried out with changing coil outer diameters of sensor as shown in Table 3.

Figure 6 shows the amplitude curve of sensor output signals for variable outer diameters. Table 4 lists the distance between separated maximum values of output signal for variable outer diameters. The experimental results indicate that the distance between separated maximum values increased with the increase of the coil outer diameter. The ratio of the max-to-max distance to the coil outer diameter was approximately 1.0. When the coil outer diameter was 10 mm, the distance between separated maximum values was 10.3 mm, the ratio of diameter to max-to-max distance was 0.97; when the coil outer diameter was 15 mm, the distance between separated maximum values was 15.4 mm, the ratio of diameter to max-to-max distance was 0.97. When the coil outer diameter of sensor was 20 mm, the distance between separated maximum values was 19.6 mm, and the ratio of the diameter to the max-to-max distance was 1.02.

**Figure 8:**
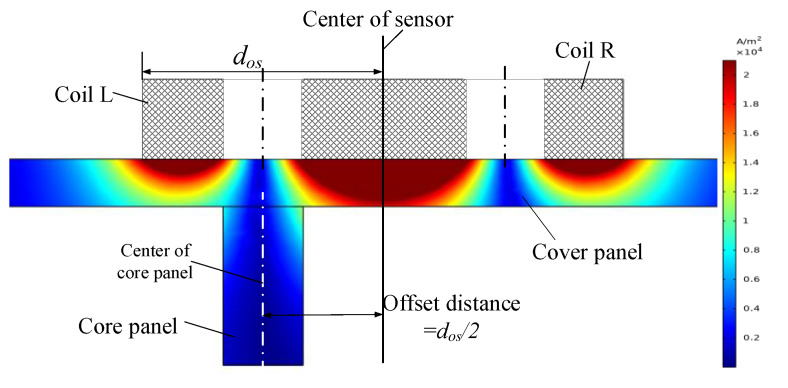
Schematic diagram while the offset distance between the centers of sensor of core was *d_os_*/2.

**Figure 9:**
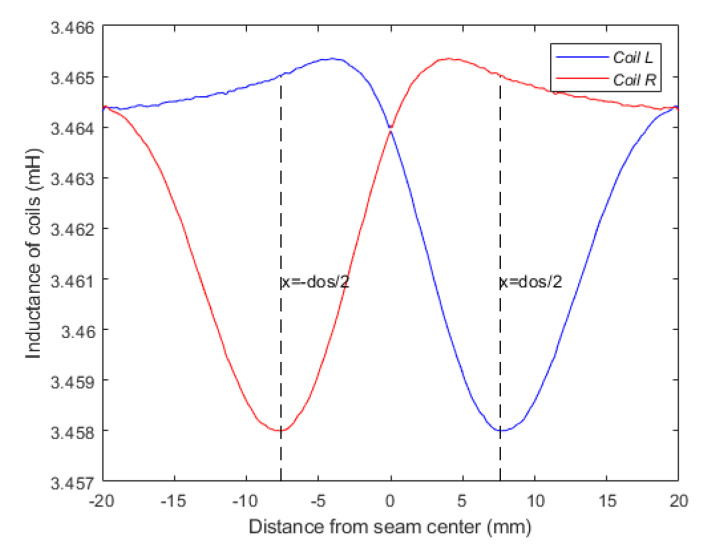
The inductance of separated coils of sensor with a different position of the weld (*d_os_* = 15 mm).

As shown in Figure 8, the center of the core panel was aligned with the center of the coil L of the sensor while the offset distance between the center of sensor and core panel was 0.5 × *d_os_*. The sensitive volume of the specimen within the detection range of the coil L reached the maximum. Thus, the effect of the specimen on the equivalent impedance of the coil L also reached the maximum, which led to the maximum difference between equivalent impedances and voltage of coil L and coil R. Figure 9 shows the inductance curves of the separated coils of sensor during single scanning of the weld seam.

**Figure 10:**
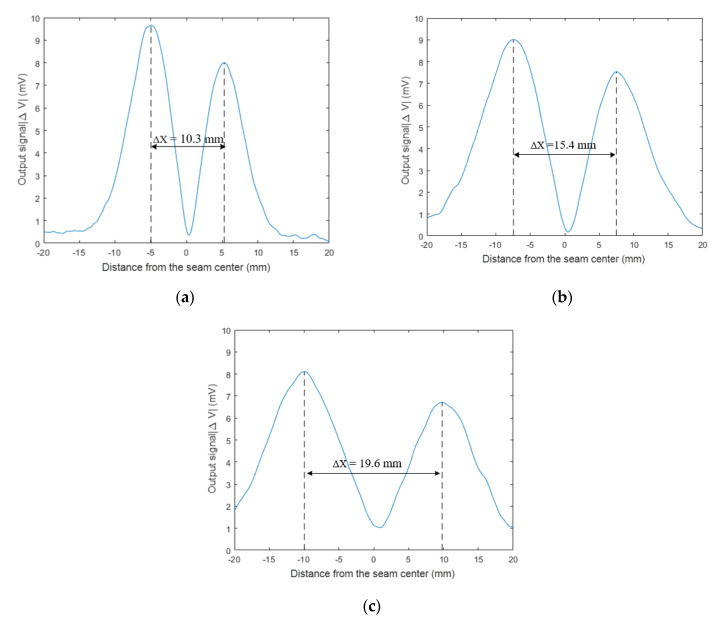
Change of relationship between the sensor-output voltage Δ*V* and coil outer diameter (**a**) 10 mm; (**b**) 15 mm; and (**c**) 20 mm.

As shown in Figure 10, with the increase of the coil outer diameter, the hump widths on both sides of the sensor output signal increased gradually. The radial detection range of the eddy current sensor is proportional to the outer diameter of the coil, and the radial sensitive range of the sensor is determined by the outer diameter of the coil [25].

In summary, the distance between the separated maximum values of the output signal in a single scanning period is determined by the coil outer diameter of sensor. The other parameters such as inductance and resistance have little influence on the max-to-max distance. Compared with the electrical parameters, the dimensions of sensor coils are easier to keep consistent in manufacturing process of different coils, which is helpful to ensure the symmetry of maximum values and detection stability of the proposed method. The reduction of coil outer diameters not only increases the sensitivity but also weaken the anti-noise performance of the detection. Therefore, it is necessary to comprehensively consider the outer diameter of the coil according to the noise level and dynamic requirements during the detection process. The sensors with smaller outer diameter coils should be selected in the actual detection process on the premise of the anti-noise performance. The smaller sensors would be helpful to shorten the scanning distance along the cross section of the weld and increase the scanning frequency. The higher scanning frequency would increase the number of feature points obtained in unit time and improve the recognition accuracy of whole weld seam.

### 3.3. Lift off Distance h

Lift-off effect is one of the most influential factors to be overcome in eddy current testing. In this paper, several experiments with different lift-off distances from 0 to 2 mm are performed.

**Figure 11:**
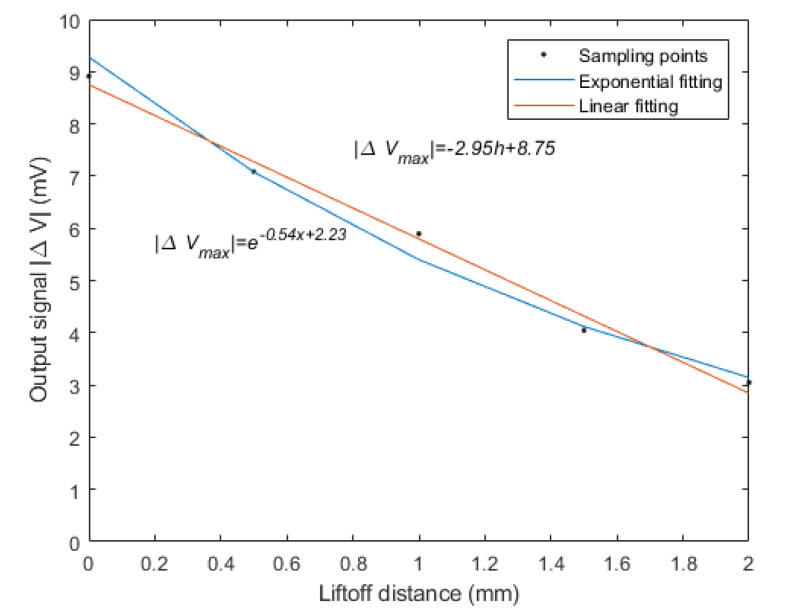
The fitting relationship between the lift off distance and the maximum value of the output signal.

As the lift off distance increased from 0 to 2 mm, the maximum values of the output signal decreased from 8.9 to 3.1 mV. As shown in Figure 11, the exponential function and linear function were respectively utilized to fit the relationship between the lift off distance and the maximum value of sensor output signal. The mean squared error of the exponential fit was 0.08 while the mean squared error of the linear fit was 0.04. The linear fit was more accurate than the exponential fit.

Keeping the other influence parameters of the eddy current sensor fixed, the output voltage *V_l_* and *V_r_* of coil L and coil R could be regarded as a single-valued function of the lift off distance *h*. The relationship between the coil voltage and the lift off distance within a small range is nearly linear. The relationship could be expressed as:

*V_l_* = *K*_1_*h*. (4)

*V_r_* = *K*_2_*h*. (5)

where *V_l_* is the output voltage of coil L; *K*_1_ is the influence coefficient of the lift off distance on the coil L alone, *h* is the lift off distance; *V_r_* is the output voltage of coil R; and *K*_2_ is the influence coefficient of the lift off distance on the coil R alone.

Therefore, the relationship between the sensor output signal and the lift off distance is nearly linear:

|Δ*V*| = |*V_l_* − *V_r_*| = |*K*_1_*h* − *K*_2_*h*| = |*K*_1_ − *K*_2_|*h*. (6)

### 3.4. The Dimensions of Specimen

#### 3.4.1. Thickness of the Core Panel *w_c_*

As one of the dimensional parameters of the weld, the thickness of the core panel is also a primary factor affecting the output signal of the sensor. In this paper, several experiments were carried out on joints with different thicknesses of core panel, and the output signals of sensor were tested. The specimen consisted of a 3 mm-thick TC4 titanium alloy cover panel and TC4 titanium alloy core panel with different thickness (5 mm, 7 mm, and 10 mm).

As the thickness of core panel increased from 5 to 10 mm, the maximum value of the output signal gradually increased from 8.9 to 33.7 mV. Due to the increase of the thickness of the core panel, the sensitive volume of the core panel within the detection sensitive area of the eddy current sensor increased significantly. Thus, the thicker thickness of core panel would lead to the increase of detection sensitivity of the sensor while the other influence parameters of the sensor kept fixed.

#### 3.4.2. The Gap Distance *g*

The gap distance is an important process parameter, which is critical for the quality of the weld. Ideally, the gap distance should be kept constant during the welding process. Nevertheless, considering assembly deformation of specimen and welding deformation during the welding process, the gap distance would change in the process. The fluctuation of the gap distance will adversely affect the quality of the weld.

Several experiments with different gap distances were carried out in this paper. The gap distance varied from 0 to 2 mm at a spacing of 0.5 mm.

**Figure 12:**
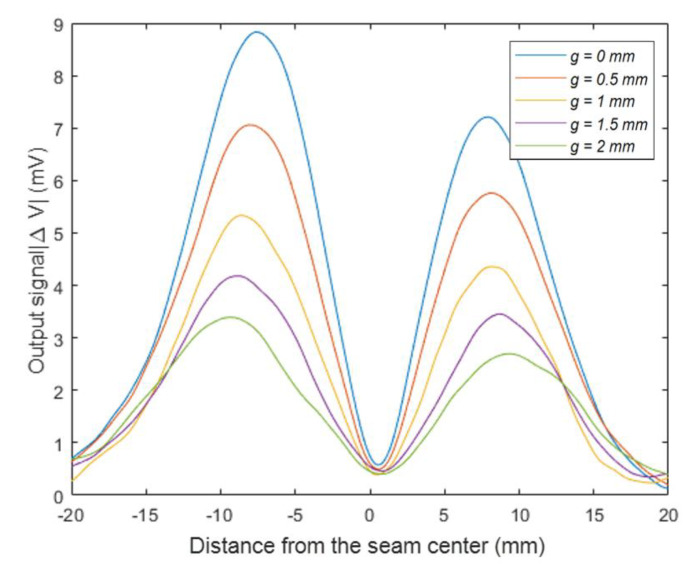
The sensor output signal with a variable gap distance.

Figure 12 shows the amplitude curves of sensor output signal under variable gap distances. As the gap distance increased from 0 to 2 mm, the maximum values of the output signal were reduced from 8.9 to 3.3 mV. In the meanwhile, the distances between the separated maximum values increased from 15.4 to 19.1 mm.

#### 3.4.3. Thickness of the Cover Panel *d*

Several experiments were carried out on the specimen with different thicknesses of cover panel. The face panel was made of TC4 titanium alloy with a thickness varies from 3 to 5 mm, while the core panel was made of 5 mm-thick TC4 titanium alloy.

**Figure 13:**
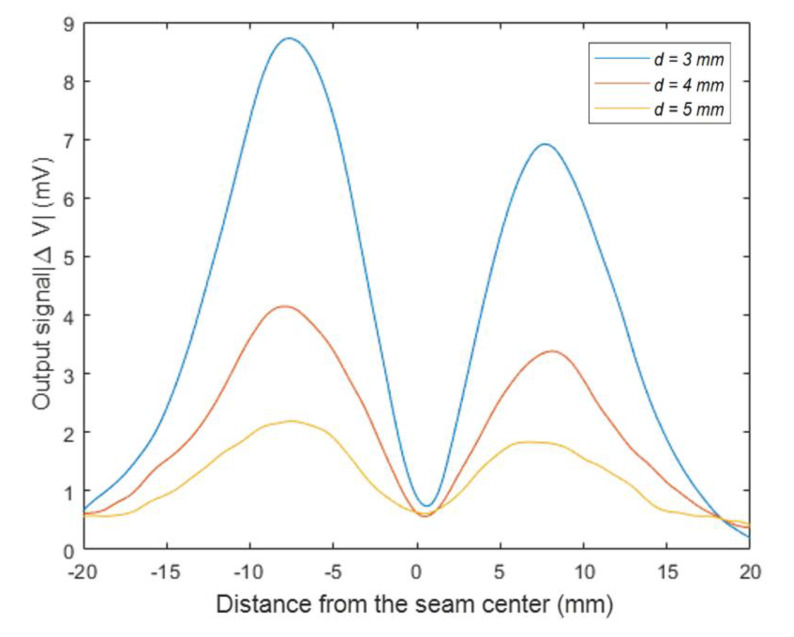
The sensor output signal with a variable thickness of the cover panel.

Figure 13 shows the amplitude curves of sensor output signals under different thicknesses of the cover panel. The maximum values of the sensor output signals decreased from 8.9 to 2.1 mV with the increase of thickness of cover panel from 3 to 5 mm, the detection sensitivity also decreased. With the same increase, the cover panel thickness had stronger attenuation effects than the gap and liftoff distance on the output signal.

**Figure 14:**
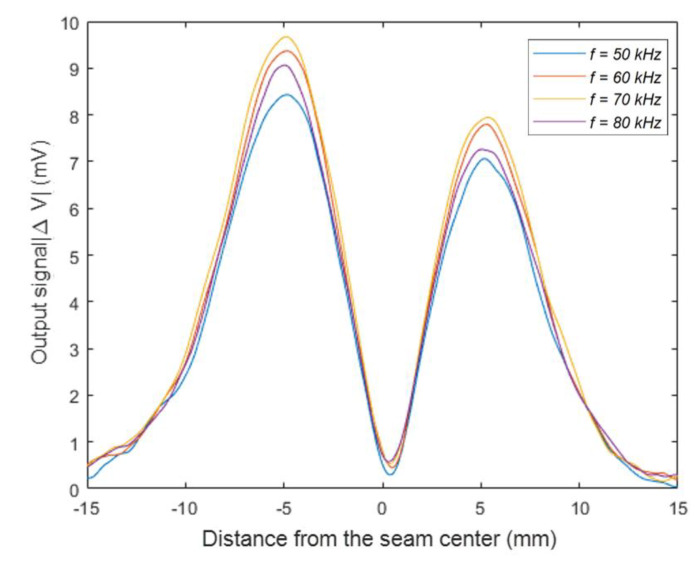
The sensor output signal at different excitation frequencies with a 2 mm-thick cover panel.

According to the Equations (2) and (3), we concluded that the optimal excitation frequency was not only related to the electrical conductivity and magnetic permeability of the specimen, but also inversely proportional to the square of the thickness of the cover panel. The effect of different material properties on the optimal excitation frequency was verified above on an aluminum/titanium alloy specimen with the same thickness of the cover panel. To verify the influence of the thickness of the cover panel on the optimal excitation frequency, we changed the thickness of cover panel and performed the detection experiments on the specimen with different excitation frequency. The specimen consisted of a 2 mm-thick TC4 titanium alloy cover panel and a 4 mm-thick TC4 titanium alloy core panel. The gap distance was 0 mm. The coil outer diameter was 10 mm. The excitation frequency varied from 50 to 80 kHz. The lift-off distance was kept 0 mm. As shown in Figure 14, the detection sensitivity reached the peak at the frequency 70 kHz. According to the Equation (2), the penetration depth reached 2.21 mm, which was close to the thickness of the cover panel.

## 4. Experimental System for Weld Detection

**Figure 15:**
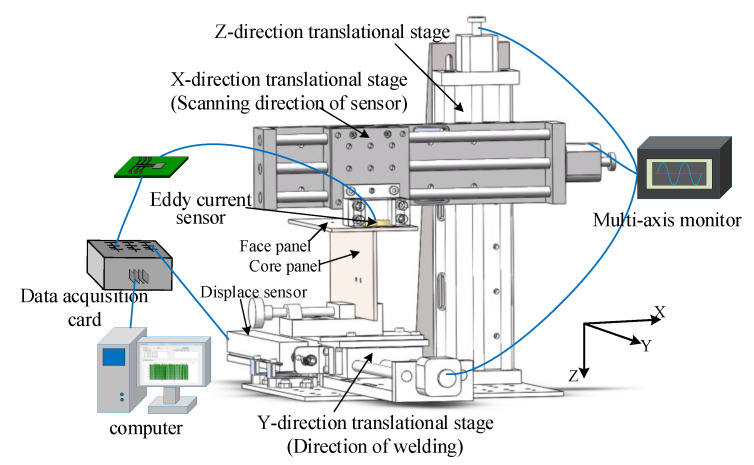
Schematic diagram of the experimental system.

In order to verify the validity of the proposed detection method, an eddy current detection system was designed. The schematic diagram of the experimental apparatus is shown in Figure 15. It consisted of an eddy current sensor, a three-axis moving platform controlled by a multi-axis monitor, the detection circuit, and a computer.

The eddy current sensor was mounted on the X-direction translational stage to scan the specimen along the cross section of weld. The specimen consisted of a face panel and a core panel, forming a T-joint as shown in Figure 15. The T-joint specimen was placed on the Y-direction translational stage to move along the welding line. The X-direction translational stage was fixed at the Z-direction translational stage to adjust the lift-off distance of the eddy current sensor and the specimen. The X–Y–Z direction translational stages formed the three-axis moving platform. The parameters of the translational stages are listed as Table 5. The output signal of eddy current sensor from the detection circuit and the signal of displacement sensor along the welding direction were fed into the data acquisition card (DAC) implemented in the computer, the parameters of the DAC are listed as Table 6. The computer was responsible for data processing and storage to obtain the characteristic value of the acquired signal, which represents the weld line.

During the detection process, the specimen was moved at a constant speed along the Y-axis direction while the eddy current sensor scanning the specimen back and forth along the X-axis at a certain frequency to acquire the feature points of the weld trajectory, and the offset from the real seam center was obtained.

## 5. Weld Detection Experiments

The experimental parameters were as follows: The eddy current sensor consisted of two separated coils with 15 mm outer diameter and 820 turns. The excitation frequency was 32.5 kHz, the lift off distance between the eddy current sensor and specimen was 0. The specimen consisted of a face panel and a core panel, forming a T-joint as shown in Figure 5. The face panel was made of a 3 mm-thick TC4 titanium alloy that was 200 mm long and 100 mm wide, while the core panel was made of 5 mm-thick TC4 titanium alloy with 200 mm length and 50 mm width, the gap distance between the cover panel and the core panel was 0. The scanning speed of the sensor along the cross section of the weld was 40 mm/s.

**Figure 16:**
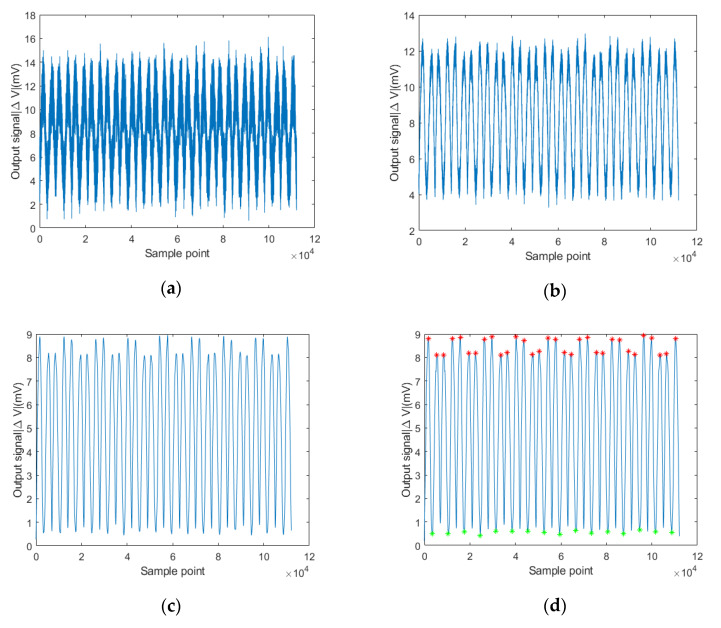
The procedure of signal processing: (**a**) original signal; (**b**) wavelet analysis; (**c**) smooth filter; and (**d**) acquisition of feature points.

The amplitude curve of the acquired detection signal is shown in Figure 16a. Since the detection signal was a small signal with relatively strong interference, the denoising algorithm was performed on the acquired signal based on wavelet analysis and smooth filter, as shown in Figure 16b,c. The characteristic values that represent the position of the weld were abrupt signals such as the maximum value or the minimum value. The wavelet analysis method was suitable for obtaining the signal mutation. The waveform of the processed signal was also retained well. It can be seen from Figure 16b that the non-stationary random noise contained in the original signal was significantly eliminated by the wavelet-based algorithm.

As shown in Figure 16, the acquired signals were processed by the proposed processing method. The feature points of weld were determined by the minimum value method and the separated maximum values method, respectively. Comparing the obtained feature points with the real seam center, the detection errors were obtained.

**Figure 17:**
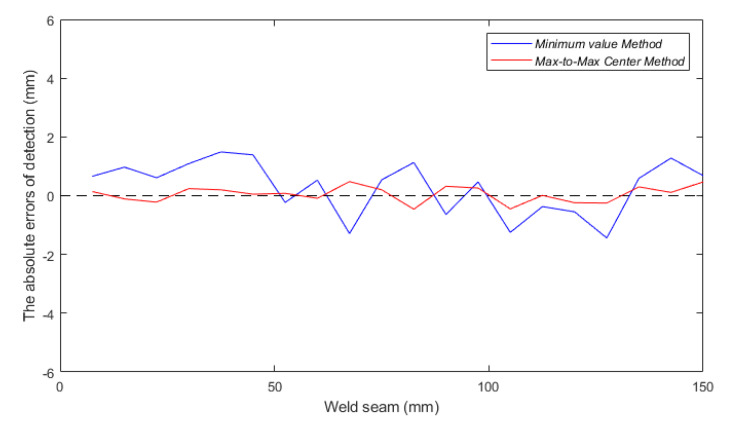
The errors of detection in the experiments.

Figure 17 and Table 7 show the detection errors of the weld feature points determined by the minimum value method and center of separated maximum values method. The blue curve indicates the detection errors of the obtained feature points of weld trajectory through the minimum value of sensor output signal. The maximum value of the errors was 1.49 mm, the average absolute error was 0.861 mm. The red curve indicates the errors of the obtained feature points of the weld trajectory through the max-to-max center method. The maximum value of the errors was 0.48 mm, the average absolute error was 0.234 mm. While welding the T-joint from the face-panel side in the sandwich structures, the maximum allowable deviation within the ±0.5 mm would be adequate for a core panel with a thickness of 5 mm.

Compared to the determination method based on the minimum value, the method based on the center of separated maximum values was more accurate. The inductance and resistance of the two separated coils of the eddy current sensor were slightly inconsistent during the manufacturing process. That results in the increase of offset of feature points determined by the minimum value. On the other hand, the random interference may occur to the detection signal due to dark noise of the detection circuit and the vibration of the mechanical scanner during the detection process. Considering the mechanical vibration is one kind of reciprocating action with a high frequency, the influence of the mechanical scanner on the detection errors of the two symmetrical maximum values would be attenuated while the weld position is determined by the max-to-max center method. That also results in the detection errors through minimum value method are higher than center of separated maximum values. As described in Section 3.2, the location of maximum values was determined by the coil outer diameter instead of the inductance and resistance. The determination method of the feature points by the difference between the separated maximum could effectively eliminate the errors caused by inconsistent inductance and resistance of sensor coils.

## 6. Conclusions

In view of the safety issue and applicability problems of existing technologies for detecting the weld position of T-joints from the face panel side of web-core sandwich panels, the paper proposed and investigated a weld position detection method based on eddy current technology. A double-coil self-comparison eddy current sensor was utilized to obtain the weld position through scanning the specimen.

The paper analyzed the characteristics of the proposed eddy current sensor. This paper investigated the relationship between the characteristics of the sensor output signal and the parameters of the detection system (including excitation frequency, coil outer diameter, and lift off distance) and the primary dimensional parameters of specimen (including thickness of core panel, gap distance, and thickness of cover panel).

(1) This paper proposed an estimation method about optimal excitation frequency to achieve the maximum detection sensitivity for a specific specimen, which could help quickly determine the corresponding detection process parameters;

(2) The coil outer diameter of the sensor determined the position of the maximum value of sensor output signal. On the one hand, under the premise of ensuring the anti-noise performance of the sensor, the scanning frequency could be increased by reducing the coil outer diameter. On the other hand, this guaranteed the accuracy of feature points of weld position determined by the max-to-max center method;

(3) The increase of gap distance would not only reduce the detection sensitivity, but also increase the distance between the separated maximum values of the acquired signals. Through analyzing the influence of above parameters on the sensor output signal, this paper provided the basis for the optimization of detection process and the extraction of characteristic values, which represent the weld position.

Finally, we designed an experimental system for the weld position detection of T-joints from the face panel side. Experiments were carried out on a TC4 titanium alloy specimen with 3 mm-thick face panel and 5 mm-thick core panel. The detection errors of feature points respectively determined by different methods were obtained. The maximum value of the detection errors based on determination method of max-to-max center was 0.482 mm and the average error was 0.234 mm. The experiments indicated that the proposed detection method based on eddy current technology was capable of meeting the accuracy requirements of weld position detection of T-joints.

## Figures and Tables

**Table 1:** The parameters of the excitation signal for simulation.

Parameter	Values
The amplitude of signal (V)	1.5
The frequency of signal (kHz)	20–40
The type of signal	sinusoidal

**Table 2:** The primary material parameters for simulation.

Material	Relative Permeability	Conductivity (S/m)	Relative Dielectric Constant
Aluminum	1	2.265 × 10^7^	1
Titanium	1	7.047 × 10^5^	1
Copper	1	5.998 × 10^7^	1

**Table 3:** The primary coil parameters.

Coil Type	Coil Outer Diameter (mm)	Coil Turns	Wire Diameters (mm)
A	10	820	0.15
B	15	820	0.15
C	20	820	0.15

**Table 4:** The distance between separated maximum values for different sensors.

Coil Type	Coil Outer Diameter (mm)	The Distance between Separated Max Values (mm)
A	10	10.3
B	15	15.4
C	20	19.6

**Table 5:** The parameters of translational stages.

Parameter	Value	Unit
Motor type	Stepping motor	-
Step angle	1.8	°
Working current	2.4	A
Motor torque	1	N·m
Backlash	≤5	um
Repeatability	≤±3	um
Load	50	kg

**Table 6:** The parameters of the data acquisition card (DAC).

Parameter	Value	Unit
Max sampling rate	500k	sps
Resolution	16	bit
Differential input channel	32	-
Programmable gain	1,2,4,8	times
Range	±5	V

**Table 7:** Comparison of detection errors from different determination methods.

Determination Method	Max Absolute Error (mm)	Average Error (mm)	Standard Deviation (mm)
Minimum value	1.49	0.861	0.542
Center of separated maximum values	0.48	0.234	0.206
